# Widespread Natural Occurrence of Hydroxyurea in Animals

**DOI:** 10.1371/journal.pone.0142890

**Published:** 2015-11-24

**Authors:** David I. Fraser, Kyle T. Liu, Bryan J. Reid, Emily Hawkins, Andrew Sevier, Michelle Pyle, Jacob W. Robinson, Pierre H. R. Ouellette, James S. Ballantyne

**Affiliations:** 1 Department of Integrative Biology, University of Guelph, Guelph, Ontario, Canada; 2 Durham College, Oshawa, Ontario, Canada; Boston University, UNITED STATES

## Abstract

Here we report the widespread natural occurrence of a known antibiotic and antineoplastic compound, hydroxyurea in animals from many taxonomic groups.

Hydroxyurea occurs in all the organisms we have examined including invertebrates (molluscs and crustaceans), fishes from several major groups, amphibians and mammals. The species with highest concentrations was an elasmobranch (sharks, skates and rays), the little skate *Leucoraja erinacea* with levels up to 250 μM, high enough to have antiviral, antimicrobial and antineoplastic effects based on *in vitro* studies. Embryos of *L*. *erinacea* showed increasing levels of hydroxyurea with development, indicating the capacity for hydroxyurea synthesis. Certain tissues of other organisms (e.g. skin of the frog (64 μM), intestine of lobster (138 μM) gills of the surf clam (100 μM)) had levels high enough to have antiviral effects based on in vitro studies. Hydroxyurea is widely used clinically in the treatment of certain human cancers, sickle cell anemia, psoriasis, myeloproliferative diseases, and has been investigated as a potential treatment of HIV infection and its presence at high levels in tissues of elasmobranchs and other organisms suggests a novel mechanism for fighting disease that may explain the disease resistance of some groups. In light of the known production of nitric oxide from exogenously applied hydroxyurea, endogenous hydoxyurea may play a hitherto unknown role in nitric oxide dynamics.

## Introduction

Hydroxyurea is a remarkable compound that has been known to science since1869 when it was first synthesized [1]. Various studies show it has antiviral, antibacterial, and antineoplastic properties [2]. Its mechanism of action involves inhibition of ribonucleotide reductase (EC 1.17.4.1) which inhibits DNA synthesis [3] in a variety of organisms. It is, or has been used in the treatment of a variety of neoplastic diseases, sickle cell anemia, psoriasis, myeloproliferative diseases and infectious diseases such as HIV [2]. It is listed as an “essential medicine” by the World Health Organization [4]. Hydroxyurea, however, is virtually unknown in nature with a record of its presence in the bacterium *Streptomyces garyphalus* as an intermediate in cycloserine synthesis [5] and a report in human plasma at levels close to the limits of detection (2.6 μM) [6]. We examined the levels of hydroxyurea in tissues of representative of invertebrate and vertebrate groups.

## Materials and Methods

### Animals

Animals collected in Passamaquoddy Bay, New Brunswick, Canada were collected under Department of Fisheries and Oceans Canada permit number 323401. Euthanasia procedures for this specific study were approved by University of Guelph Animal Care Committee Protocol number 11R014. Surf clams, *Spisula solidissima*, were collected at low tide at Bar Road, St. Andrews, New Brunswick Canada. Lobsters, *Homarus americanus* were purchased from a local (Guelph, Ontario, Canada) seafood retailer. Hagfish, *Eptatretus stouti* tissues were donated by D. Fudge, Department of Integrative Biology, University of Guelph. Little skates (*L*. *erinacea* Mitchill 1825) of either sex were collected by otter trawl in Passamaquoddy Bay (New Brunswick, Canada), before transport to holding facilities in the Hagen Aqualab, at the University of Guelph (Guelph, Ontario) where they were maintained for several months to several years. Skate eggs were obtained from this colony. African lungfish (*Protopterus dolloi*) were held and sampled as previously described [7]. Adult rainbow trout (*Oncorhynchus mykiss* Walbaum 1792) of either sex were purchased from a local fish farm (Belleville, Ontario) and transported to holding facilities at the University of Guelph. Trout were held as previously described [8,9]. Frogs, *Lithobates pipiens* tissues were donated by P. Smith, Department of Integrative Biology, University of Guelph. Sheep, Ovis aries, tissues were obtained from a local slaughterhouse (Guelph, Ontario, Canada). Animals collected in Passamaquoddy Bay, New Brunswick, Canada were collected with permission of the Department of Fisheries and Oceans Canada permit number 323401.

### Sampling

Fish were euthanized by cervical section. Tissues were rapidly excised, frozen in liquid nitrogen and stored at –80°C until used. Blood was drawn by cardiac (skates) or caudal (other fish) puncture using heparinized syringes. Erythrocytes were separated from plasma by centrifuging blood at 2,430 *g* for 10 minutes at 4°C. Sheep tissues were collected from a federally regulated abattoir at the University of Guelph.

### Preparation of tissues for use in hydroxyurea and urea assays

Tissues were homogenized in a small volume of ddH_2_O using a Polytron PT1200 homogenizer set at high speeds (25, 000 rpm) for three 10 second bursts, with a cooling period of 30 seconds between each burst. Homogenized samples were then spun at 9,700 *g* with a Sorval SA-600 rotor and 4°C for 10 minutes to remove cellular debris. The resulting supernatants and diluted plasma samples were collected and deproteinized with 60% perchloric acid (PCA) to a final concentration of 0.5M PCA. Acidified samples were then spun at 22,000 *g* for 20 minutes with a Sorval SA-600 rotor and 4°C. The supernatants were collected for use in hydroxyurea and urea assays and the pellets discarded.

### Measurement of hydroxyurea and urea in biological samples

**Fig 1:**
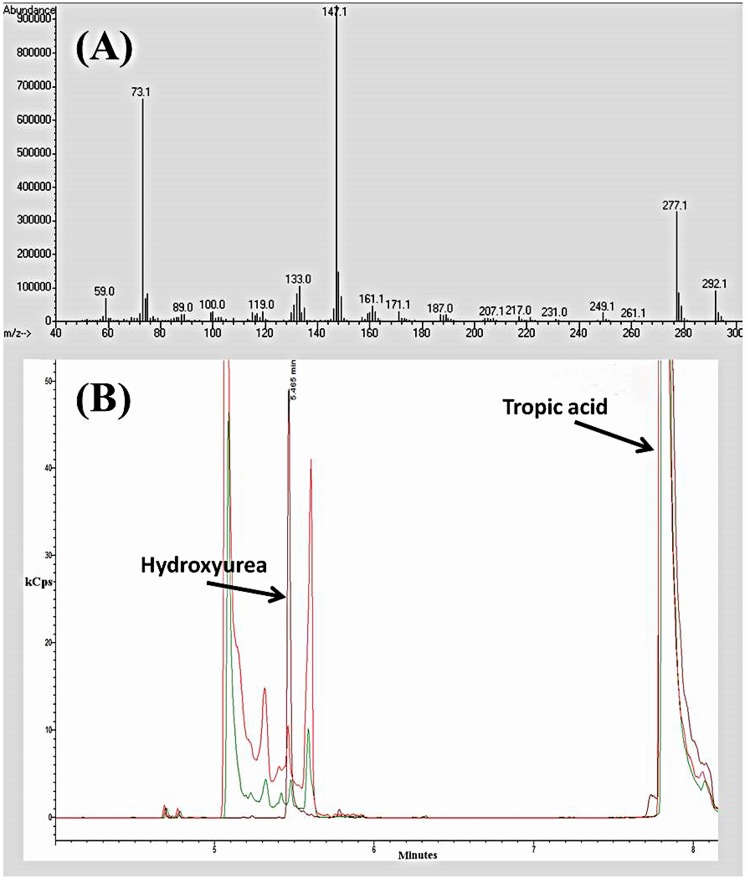
A: Fragmentation pattern for derivatized hydroxuyrea (hydroxyurea triTMS). B: Overlaid GC-MS chromatograms of a 10 μg/ml standard of hyroxyurea (maroon), deproteinized liver sample from *L*. *erinacea* (green), and deproteinized plasma sample from *L*. *erinacea* (red). Peaks at elution time ~5.4 min represents hydroxyurea while peaks at ~ 7.8 min represents tropic acid (10 μg/ml) an internal standard in all three samples. Chromatograms were selective for ions 277 (representative of hydroxyurea triTMS) and 280 (representative of tropic acid-diTMS). X-axis = minutes, Y-axis = kCps.

Determination of hydroxyurea content in deproteinized plasma and tissue samples followed the colorimetric assay of Fabricius and Rajewsky [10]. Absorbance of hydroxyurea samples was measured at 540 nm using a Cary 300 UV/Vis spectrophotometer (Agilent Technologies). Urea was measured according to the protocol originally described by Rahmatullah and Boyde [11] at 525 nm. In our study, hydroxyurea was measured chemically by the method of Fabricius and Rajewsky [10] with analate addition. Its identity was confirmed by gas-chromatography mass spectrometry using the method of Scott et al. [12] in plasma and liver samples from *L erinacea* (Fig 1).

### Gas Chromatography-Mass Spectrometry (GC/MS)

Samples were derivatized as detailed in Scott et al. [12]. Once derivatized, tubes were cooled to room temperature and the contents transferred to autosampler vials containing tapered inserts before being placed in the autoinjector of the GCMS and run. Injections of 1 ml were used for GCMS analysis. GC/MS operating conditions were adapted from those detailed in Table 26.3 of Scott et al. [12]. The GC/MS was operated in selected ion mode after electron impact fragmentation, selective for ion 277 (hydroxyurea tri-TMS).

### Statistics

ANOVA with a Tukey Post Hoc Test was conducted to identify significant differences (P<0.05) in HU or urea concentrations between tissues.

## Results and Discussion

**Fig 2:**
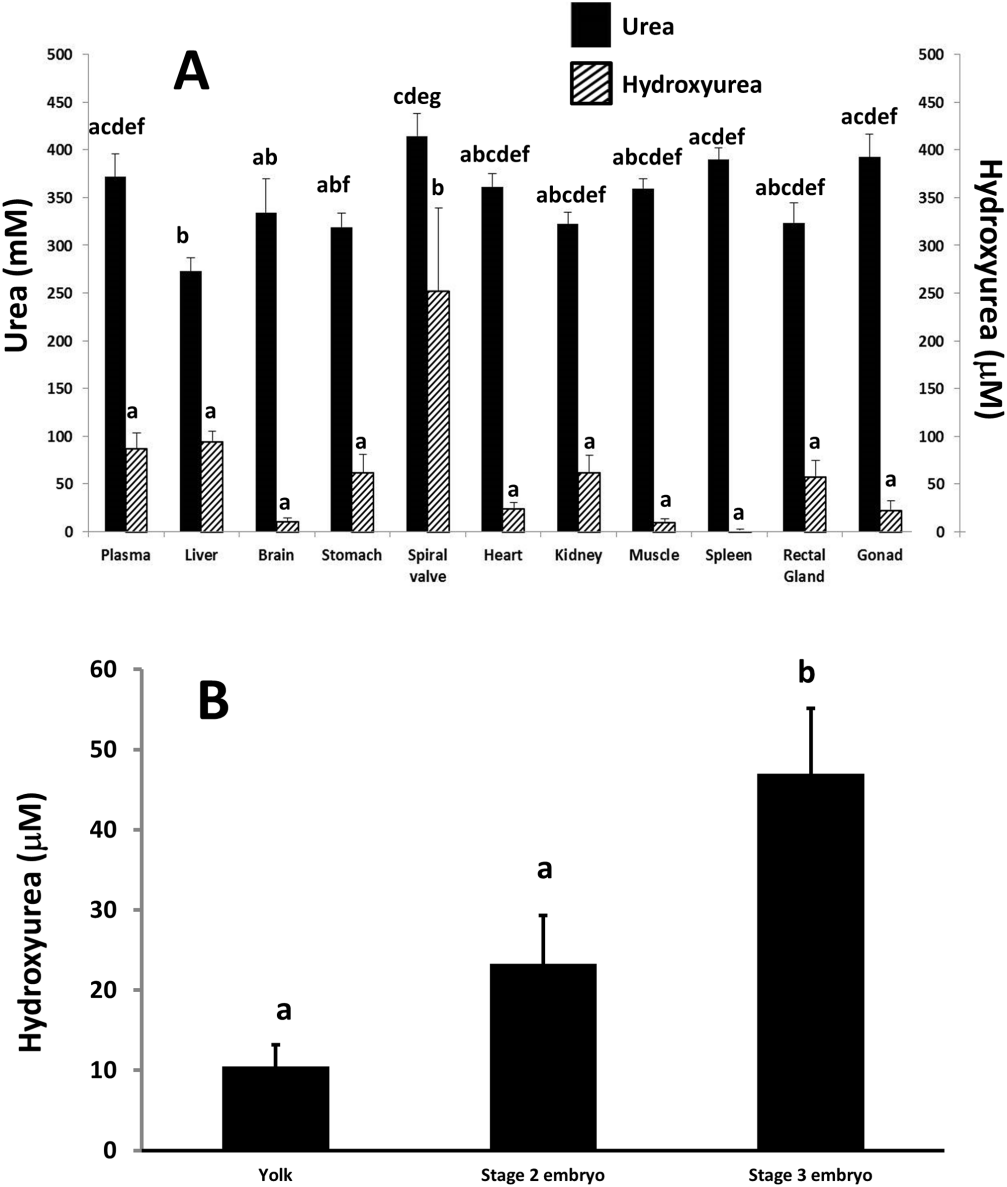
A: The distribution of hydroxyurea and urea in the blood plasma and tissues of adult little skates *(L*. *erinacea*, n = 5). Hydroxyurea was non-detectable in erythrocytes. Values are means ± standard error (SE) of the mean. Values with the same letter above the bar are not significantly different from each other. B: Whole body hydroxyurea concentrations (mean μM ± SE) measured in little skate (*L*. *erinacea*) embryos at stages 2 and 3 of development according to criteria described by Hoff [30]. Values are from whole embryos with yolk sacs removed from both stage 2 and 3 embryos. Values are means ± standard error (SE) of the mean. Values with the same letter above the bar are not significantly different from each other.

We initially found tissue specific accumulation of hydroxyurea in the elasmobranch *L*. *erinacea* with values up to 250 μM in the spiral valve (intestine) (Fig 2a). The extensive literature on the antibiotic and antineoplastic effects of hydroxyurea lead to the obvious conclusion that its biological role in animals is as part of the innate immune system to combat viral and other infections. The nominal concentrations we report for the little skate are in the range of concentrations causing 50% inhibition (ED_50_) of a variety of processes including DNA synthesis, ribonucleotide reductase activity in viruses and growth of some cell types (Table 1). The use of the values presented in Table 1 in comparison to the values we report here has several caveats that must be considered. Firstly, the times used for determination of the ED_50_ reported in Table 1 range from 10 minutes to several days in vitro. However, according to Haber’s law, the severity of a toxic effect depends on the total exposure (i.e. exposure concentration multiplied by the duration of exposure) [13]. Maintenance of chronic high levels of hydroxyurea *in vivo* would thus reduce the concentration needed for a given effect. Thus, the hydroxyurea levels we report would be even more effective *in vivo* than the values in Table 1 would predict. Secondly, our hydroxyurea tissue concentrations are likely to be underestimates in the vertebrates we examined since hydroxyurea reacts with hemoglobin and some would be destroyed during preparation in tissues with substantial blood supplies [14]. Concentrations in the spleen especially may be higher than measured since, as a storage site for erythrocytes the spleen has the highest concentrations of hemoglobin of any tissue.

**Table 1:** Effective dose levels of hydroxyurea at which 50% of a process is inhibited (ED_50_). Values are reported in either micromolar (μM) or millimolar (mM).

Process	ED_50_	Time	System	Reference
DNA synthesis	50 μM	3 hours	HeLa cells	[15]
	100 μM	30 min	He La cells	[16]
	132 μM	1 hour	Ascites tumor cells	[17]
	200 μM	48 hours	*Chlamydia trachomatis*	[18]
	250 μM	24 hours	Murine mastocytoma cells P815, human myelogenous leukemia K562 cells	[19]
	1 mM	48 hours	*Chlamydia trachomatis*	[18]
Ribonucleotide reductase	10–30 μM	10 min	T4 phage	[20]
	150–160 μM	10 min	*Escherichia coli*	[20]
	1 mM	24 hours	Murine leukemia cells	[19]
Growth	19.7 μM	45 hours	Chinese hamster cells	[21]
	65 μM	45 hours	HeLa cells	[21]
	180 μM	72 hours	A549 lung carcinoma cells	[22]
	2.5 mM	18 hours	*Pseudomonas aeruginosa*	[23]
Viral replication	60 μM	72 hours	Hepatitis C in OR6 cells	[24]
	75 μM (from Fig 3c of reference)	7 days	HIV virus	[25]
	100 μM (from Fig 1c of reference)	Not given	HIV virus	[26]
	1 mM	1 week	*Vaccinia* in Chinese hamster cells	[27]
Survival	2 μM	6 days	*Leishmania mexicana*	[28]
	400 μM	1 hour	Chinese hamster cells	[29]

The values we report for *L*. *erinacea* are in the range that would affect some viral and bacterial processes (Fig 2a and Table 1). Generally, viral processes are more susceptible to inhibition by hydroxyurea than bacterial or mammalian cell lines (Table 1) [31]. Interestingly, our plasma concentrations for *L*. *erinacea* (87 μM) correspond to the range maintained to treat human HIV Type 1 patients (10–130 μM): the range that inhibits HIV in vitro [32].

Elasmobranchs are an ancient vertebrate group with unusual physiological and biochemical characteristics [33]. They are the earliest known vertebrate group to have an adaptive immune system using antibodies. Among the features of their biology that has attracted public interest is their anecdotal resistance to disease, especially cancer. There is little hard science to validate such claims but the available literature records few viral and bacterial diseases from this group in spite of a considerable interest [34]. Among vertebrates, the incidence of neoplasia is lowest in elasmobranchs [35,36]. Reports of the unusual occurrence of bacteria in plasma [37] and tissues [38] of apparently healthy elasmobranchs may also be due to the effects of hydroxyurea in preventing bacterial growth.

Although the mechanism of synthesis of hydroxyurea *in vivo* is not currently known, the presence of hydroxyurea in embryos from eggs of *L*. *erinacea* and the increase in concentration as the embryo grows (Fig 1b) provides evidence of the capacity for hydroxyurea synthesis in *L*. *erinacea*.

**Fig 3:**
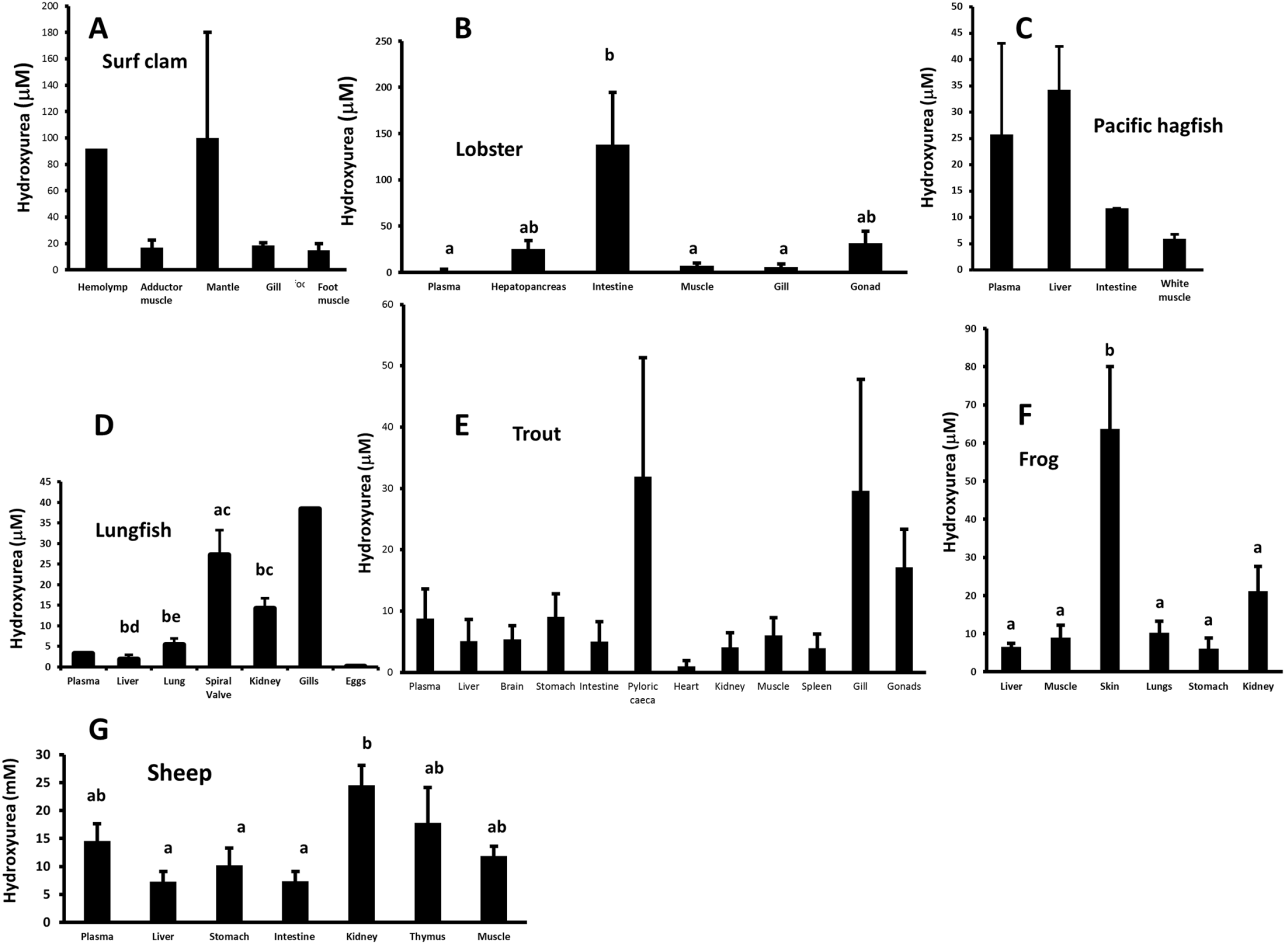
Hydroxyurea levels in tissues of A) the surf clam *S*. *solidissima* n = 1–3); B) American lobster, *H*. *americanus* n = 5–6; C) Pacific hagfish *E*. *stouti*, n = 2–4; D) African lungfish, *P*. *annectens*, n = 2–7; E) rainbow trout, *O*. *mykiss*, n = 6; F) frog, *L*. *pipiens*, n = 6; G) the sheep, *O*. *aries*, n = 5–6. Values are means ± standard error (SE) of the mean. Values with the same letter above the bar are not significantly different from each other. Tissues of the clam were not included in the statistical analysis due to the low n values. There were no differences between tissues of the Pacific hagfish or trout. Due to the low n value for plasma, gill and eggs of the lungfish is these tissues were not included in the analysis and have no letter above the bar.

Levels of hydroxyurea in tissues of other species including invertebrates and vertebrates are for the most part lower than those of the little skate (Fig 3a–3g). Similar to the situation in the skate, the distribution is tissue specific. In the surf clam, *S*. *solidissima* levels were high in mantle (100 μM) and in the lobster, *H*. *americanus* high levels were found in the intestine (138 μM). In the non-elasmobranch vertebrates, levels were generally low with the highest levels being found in skin (64 μM) of the frog *L*. *pipiens*. In the lungfish, *P*. *annectens*, the highest levels were found in the gills (38 μM). In the trout, *O*. *mykiss*, the highest levels were found in the pyloric caecae (32 μM). In the sheep, *O*. *aries*, the highest levels were in kidney (25 μM). If one assumes that endogenous hydroxyurea confers some defense against viral or other infection, significantly higher levels in some tissues may mean these are sites that need to be defended most.

The tissue specific distribution of hydroxyurea indicates either it can be synthesized locally or transported and concentrated. Its structural similarity to urea (both are polar, with a low molecular weight differing only in the presence of a hydroxyl group) suggests it could be transported by the same carriers as urea but the tissue distribution of these 2 compounds in *L*. *erinacea* is very different (Fig 2a). The main organic osmolyte of marine elasmobranchs is urea that is accumulated to levels more than one thousand times that of hydroxyurea [33] (Fig 2a). In general, tissue specific differences in urea content are small (~20%). Hydroxyurea concentrations, on the other hand can vary by as much as 25 fold between tissues. Thus there must be transporters that can distinguish urea and hydroxyurea and these are known in mammals [39]. In vitro studies show hydroxyurea is far less permeant than urea in mouse erythrocytes due to the capacity of the urea transporter B (UT-B) to distinguish between them [39]. Several carriers for hydroxyurea have been identified including the organic anion transporting polypeptides (OATP) OATP1A2, OATP1B1 and OATP1B3, organic cation transorters (OCT), OCTN1 and OCTN2 and urea transporters A and B [40]. Active transport of hydroxyurea by OCT1B3 has also been suggested [40].

An important consideration in understanding the impact of retaining high levels of hydroxyurea in tissues is its inhibitory effect of ribonucleotide reductase (RR), an enzyme needed by all cells for DNA synthesis. Mammalian RR is less susceptible to inhibition by hydroxyurea than viral or bacterial RR (Table 1). This would be important for inhibition of viral and bacterial replication without affecting mammalian cell growth. Hydroxyurea thus could provide a level of protection against viral and other challenges as part of the innate immune defense mechanism.

Our findings of hydroxyurea in a mammal, although at levels 5–10 fold lower than in the elasmobranch, may be particularly important for understanding mammalian disease resistance. Exogenously applied hydroxyurea has been shown to stimulate nitric oxide production in mammalian systems [41,42]. Nitric oxide synthase plays a key role in the killing of pathogenic organisms by phagocytes [43] although the mechanism is not known [44]. We suggest a role for naturally occurring hydroxyurea in phagocyte function via the following mechanism.

**Fig 4:**
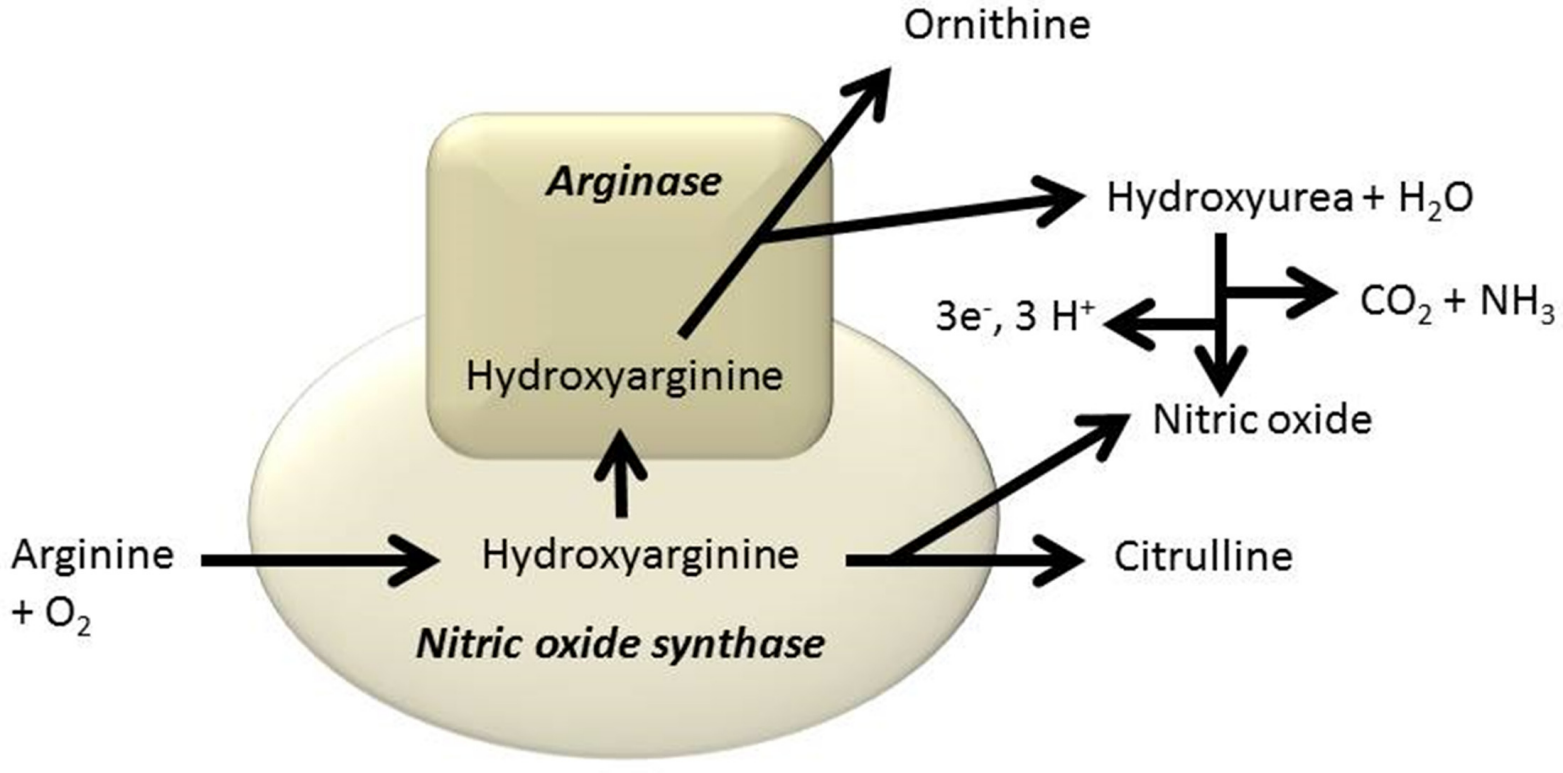
Diagram of the proposed pathway for the synthesis of hydroxyurea in animal tissues based on [49].

Nitric oxide is produced from arginine by nitric oxide synthase (NOS) via the intermediate hydroxyarginine. Arginase is known to react with hydroxyarginine *in vitro* to produce hydroxyurea instead of urea [45]. We propose that *in vivo* some hydroxyarginine is diverted to hydroxyurea synthesis by arginase and the hydroxyurea converted to NO as depicted in Fig 4. This mechanism helps explain the paradoxical colocalization of arginase and NOS in cells such as human endothelial cells [46]. In endothelial cells arginine metabolism is highly compartmentalized [47] and arginase is known to compete with NOS for arginine [48].

## Conclusions

Our finding that hydroxyurea occurs in many animal groups at levels that could act as a defense against viral and other challenges implies: a) a new component to the innate immune system of animals that may explain superior disease resistance of some groups and b) a new intermediate in the pathway for NO production in animals.
